# Serum d-serine and d-amino acid oxidase (DAO) levels in schizophrenia and related psychotic disorders: a 6-month follow-up study

**DOI:** 10.1192/j.eurpsy.2024.228

**Published:** 2024-08-27

**Authors:** E. Uzun Uysal, N. B. Tomruk, C. Çakır Şen, E. Yıldızhan

**Affiliations:** Department of Psychiatry, Bakırköy Research and Training Hospital for Psychiatry, Neurology and Neurosurgery, İstanbul, Türkiye

## Abstract

**Introduction:**

D-serine and the DAO enzyme may impact the NMDA receptor and contribute to schizophrenia, but the exact role and outcomes are not fully understood due to the complexity of the disorder.

**Objectives:**

We analyzed serum levels of d-serine and DAO in untreated individuals with schizophrenia during acute psychotic episodes. We correlated these factors with clinical characteristics and compared results to a healthy control group. We also examined any differences after six months of treatment.

**Methods:**

The study involved 89 patients with schizophrenia or related psychotic disorders who were hospitalized due to psychotic episodes. Also, the study had 81 healthy participants matched in terms of gender, age, and smoking status with the patient group. PANSS, CGI, GAS, CDSS, and MoCA were applied to determine the severity of the disease. Serum d-serine and DAO levels were measured by ELISA kits.

**Results:**

During an acute psychotic episode, patients had significantly lower levels of D-serine, DAO, and D-serine/DAO ratio compared to healthy individuals (Z=6.52, p<0.001; Z=4.54, p<0.001; Z=2.90, p=0.004). Although DAO and D-serine levels increased with symptom regression after six months of treatment, the D-serine and D-serine/DAO ratios were significantly lower in patients than in healthy individuals(Z=3.52, p<0.001; Z=3.44, p<0,001). There was no correlation between the change in D-serine level and the change in scale scores. However, there was a negative correlation between the change in DAO level and the change in PANSS total (r=-0.681, p=0.000), anxiety scores (r=-0.336, p=0.032), and Calgary depression score (r=-0.547, p=0.000). There was a positive correlation between the change in D-serine/DAO ratio and the change in the Calgary depression scale score (r=0.353, p=0.024) in addition to PANSS positive (r=0.395, p=0.011) and total scores (r=0.585, p=0.000). Antipsychotic doses negatively correlated with the changes in DAO level (r=0.421, p=0.01). It was found that the female patients had significantly lower levels of DAO than the female healthy subjects (Z=-5.061, p<0.001). No correlation was found between serum D-serine level, DAO level, and the D-serine/DAO ratio with cognitive function. D-serine level negatively correlated with age(r=-0.265, p=0.012) and age at onset of the disease (r=-0.227, p=0.032).

**Conclusions:**

The findings support the view that D-serine and DAO may play a role in the pathophysiology of schizophrenia and related psychotic disorders. To better understand the relationship between D-serine metabolism and symptom clusters in psychosis and the effects of antipsychotic drugs on NMDAR dysfunction, further studies that directly measure DAO enzyme activity and examine cognitive symptoms in more detail are needed.

**Disclosure of Interest:**

None Declared

